# Encystation of *Entamoeba histolytica* in Axenic Culture

**DOI:** 10.3390/microorganisms9040873

**Published:** 2021-04-18

**Authors:** Jordan Wesel, Jennifer Shuman, Irem Bastuzel, Julie Dickerson, Cheryl Ingram-Smith

**Affiliations:** 1 Department of Genetics and Biochemistry, Clemson University, Clemson, SC 29634, USA; jwesel@g.clemson.edu (J.W.); jhbattl@g.clemson.edu (J.S.); ibastuz@g.clemson.edu (I.B.); jadicke@g.clemson.edu (J.D.); 2 Eukaryotic Pathogens Innovation Center, Clemson University, Clemson, SC 29634, USA

**Keywords:** *Entamoeba*, *Entamoeba histolytica*, *Entamoeba invadens*, encystation, cyst

## Abstract

*Entamoeba histolytica* is a parasitic protozoan that causes amoebic dysentery, which affects approximately 90 million people each year worldwide. *E. histolytica* is transmitted through ingestion of food and water contaminated with the cyst form, which undergoes excystation in the small intestine to the trophozoite form that colonizes the large intestine. The reptile pathogen *Entamoeba invadens* has served as a model for studying stage conversion between the trophozoite and cyst form due to lack of reproducible encystation of *E. histolytica* in the laboratory. Although much has been learned about encystation and excystation using *E. invadens*, the findings do not fully translate to *E. histolytica* due to the extensive genetic and host differences between these species. Here, we present the first reproducible encystation of *E. histolytica* in vitro. The cysts produced were viable and displayed the four characteristic hallmarks: round shape, chitinous cell wall, tetranucleation, and detergent resistance. Using flow cytometry analysis, glucose limitation and high cell density were key for encystation, as for *E. invadens*. Entry into encystation was enhanced by the short-chain fatty acids acetate and propionate, unlike for *E. invadens*. This new model will now allow the further study of *E. histolytica* stage conversion, transmission, and treatment.

## 1. Introduction

*Entamoeba histolytica* is a protozoan parasite of humans that is the causative agent of amoebic dysentery. This pathogen is particularly common in tropical areas in developing nations with poor sanitation [1,2], and approximately 90 million people worldwide experience symptomatic disease from *E. histolytica* infection each year [2]. This is thought to represent just a fraction of infections though, as an estimated 90% of people infected with *E. histolytica* are asymptomatic carriers [3,4]. Thus, *E. histolytica* infection represents a major public health problem affecting up to 1 billion people yearly, resulting in significant loss of productivity and 50,000–100,000 deaths from amoebic liver abscess [5]. Amoebiasis ranks as a leading cause of death due to parasitic infections [6,7,8].

*E. histolytica* is transmitted via the oral–fecal route. Transmission occurs when *E. histolytica* is shed from the host as an infective, environmentally resistant cyst and ingested in contaminated food or water. After ingestion, the cyst emerges into its disease-causing trophozoite form in the small intestine and then colonizes the large intestine. Although most cases are asymptomatic, carriers shed millions of cysts daily and thus perpetuate the infection cycle [9]. Symptomatic infections typically present as amoebic dysentery but can also result in amoebic liver abscesses that can be deadly if left untreated [4,10,11].

Despite the global burden of this disease, laboratory study of *E. histolytica* infection has been seriously impeded by the inability to induce true encystation in the laboratory [12,13,14,15,16]. *E. histolytica* can be readily cultured in a laboratory setting in the trophozoite stage, and genetic tools exist to manipulate the parasite and study the disease-causing state. Development of a reproducible method for inducing encystation of *E. histolytica* in culture has remained intractable though. One possible explanation is that due to the nature of their continuous cultivation, the virulence of laboratory strains has been reduced, and differentiation to cysts can no longer be induced in axenic culture [12]. The lack of an animal model that can support the complete *E. histolytica* life cycle from ingestion of cysts to colonization to shedding of cysts has also hampered our understanding of the virulence, infection cycle, and phase transitions between cysts and trophozoites. Instead, researchers have relied on direct injection or surgical inoculation of trophozoites to analyze infection in the mouse and gerbil models of infection [17,18,19,20,21,22].

Previous attempts to induce *E. histolytica* encystation in laboratory culture have been unsuccessful in producing viable cysts that display the four primary hallmarks: spherical shape, detergent resistance, chitin cell wall, and tetranucleation. Said-Fernandez and Mata-Cárdenas [23] reported the formation of cyst-like structures after one or two nuclear divisions. The efficiency of the production of these cyst-like structures was ~32.5% after 7 days, similar to the results presented here, but only 1.2% were tetranucleated and could not withstand osmotic pressure. Barrón-González et al. [14] induced the formation of cystlike cells at a very high efficiency through the use of *Escherichia coli* and *Enterococcus faecalis* conditioned media and alkaline pH. These cyst-like cells were multinucleate but were typically not tetranucleate and were not viable for excystation. Aguilar-Diaz et al. [13] induced the formation of cyst-like structures after treatment with hydrogen peroxide and metals, but these structures were not consistently tetranucleate, and the maximum conversion rate from trophozoite to cyst was only ~30%.

Researchers have relied on the reptilian species *Entamoeba invadens* as the model for studying *Entamoeba* stage conversion [24,25]. The IP-1 strain of *E. invadens* encysts in just 72 h upon application of glucose deprivation and hypo-osmotic shock [26,27]. *E. invadens* IP-1 can encyst with >95% efficiency unlike other *E. invadens* strains that encyst at only 5–20% efficiency [28,29]. Although this super encyster has provided a great deal of information on encystation and excystation, the findings cannot not fully translate to *E. histolytica* due to genetic and host differences. *E. invadens* has over 3000 more predicted genes than *E. histolytica*, and less than half of the predicted genes in *E. invadens* have putative orthologs in *E. histolytica* [30]. Of 195 cyst-specific proteins identified through proteomic analysis of *E. histolytica* cysts [31], only 74 had orthologs in *E. invadens* [30].

We have now developed a method for encystation of *E. histolytica* in axenic culture. Microscopic examination demonstrated that the cysts were encapsulated in a chitin cell wall, were spherical and smaller in size than trophozoites, and were multinucleated with the majority having four or more nuclei. These cysts were also shown to be detergent resistant, thus fulfilling all four hallmarks of true cysts. Flow cytometry analysis of encysting populations revealed that the glucose limitation and high cell density were determining factors in encystation and that the presence of the short-chain fatty acids acetate and propionate also influenced entry into encystation. Finally, cysts were shown to be viable for excystation back to the growing trophozoite form. This is the first demonstration of reproducible encystation in axenic culture to produce viable cysts, thus completing the full developmental cycle of *E. histolytica* in laboratory culture. This represents a major research breakthrough for studying a human pathogen of significant public health importance.

## 2. Materials and Methods

### 2.1. Materials

Adult bovine serum was purchased from Gemini Bio (www.gembio.com (accessed on 16 April 2021)) and was heat-inactivated at 56 °C for 25 min. Other media components were purchased from Fisher Scientific and VWR. Cells were cultured in 25 cm^2^ untreated Falcon tissue culture flasks.

### 2.2. E. histolytica Culture Maintenance

The *E. histolytica* strain HM1:IMSS was used for all experiments. All media formulations used in this study are based on TYI-S-33 medium [32] and are designated as follows: TYI glucose, standard TYI-S-33 medium containing 50 mM added glucose; TYI basal, TYI-S-33 medium lacking added glucose; TYI acetate, TYI basal medium with sodium acetate added to a final concentration of 62 mM; TYI propionate, TYI basal medium with sodium propionate added to a final concentration of 24 mM; and TYI butyrate, TYI basal medium with sodium butyrate added to a final concentration of 23 mM. The pH of each medium was adjusted to 6.8 after the addition of glucose or the given SCFA. *E. histolytica* strain HM1:IMSS trophozoites were grown axenically at 37 °C in 25 cm^2^ untreated tissue culture flasks containing 52 mL medium. Cells were passed into fresh medium approximately every 3 days for standard maintenance.

### 2.3. Encystation and Sarkosyl Treatment

To induce encystation, trophozoites were seeded at 50,000 cells/mL into 25 cm^2^ tissue culture flasks containing 52 mL TYI basal medium or TYI basal medium containing acetate, propionate, or butyrate as indicated above. Cultures were incubated at 37 °C for up to 168 h unless noted otherwise. Time courses for encystation were performed using replicate cultures with one culture from each biological replicate harvested at each time point. Flasks were incubated on ice for 10 min, and cells were harvested by centrifugation at 500× *g* for 5 min. Mature detergent-resistant cysts from a 52 mL culture were purified from trophozoites by treatment with 5 mL phosphate-buffered saline (PBS) containing 0.1% sarkosyl on ice for 10 min, followed by two washes with water. Three biological replicates were performed for each time course.

### 2.4. Viability and Excystation

Cyst viability was examined using the fluorescein diacetate (FDA) hydrolysis assay [13,33]. Cysts from 168 h cultures were harvested, washed twice with PBS, and sarkosyl-treated as described above. Samples were adjusted to 1 × 10^6^ cysts/mL in PBS, and 100 μL samples were treated with 1.6 μL of 2.5 mg/mL FDA stock solution prepared in acetone. Samples were incubated at room temperature for 8 min and visualized by fluorescence microscopy using a BP350-460 filter. The percent of viable cysts was determined by counting the number of fluorescent cysts per 100 cysts in three independent experiments.

Viability was also analyzed by the ability of cysts to excyst back to the growing trophozoite form. Cyst populations treated with sarkosyl or water to disrupt trophozoites were inoculated into TYI glucose medium and incubated at 37 °C. Excystation and outgrowth to trophozoite form was monitored microscopically.

### 2.5. Staining and Fluorescence Microscopy

Propidium iodide staining. *E. histolytica* trophozoites or sarkosyl-treated cysts from a 52 mL culture were permeabilized in 1 mL 70% ethanol at 4 °C for 30 min with periodic vortexing, washed twice with PBS, resuspended in 1 mL PBS containing 0.2 mg/mL RNAse A, and stained with propidium iodide (50 µg/mL). Cells were stained for 15–20 min, rinsed once with PBS, and visualized microscopically.

WGA-488 staining. *E. histolytica* trophozoites or sarkosyl-treated cysts were stained with 1 mL of PBS containing 10 μg/mL AlexaFluor WGA-488 (1 mg/mL) for 20 min at ambient temperature with constant rotation. The cells were then rinsed with PBS and fixed in 1 mL 4% (*v:v*) paraformaldehyde for 15 min before imaging.

Imaging. Stained cells were imaged using a Leica SPE microscope. Cells stained with WGA-488 and propidium iodide were excited at 488 and 532 nm, respectively. Differential interference contrast (DIC) images were also taken.

### 2.6. Flow Cytometry Analysis

Flasks were placed on ice for 10–15 min to release cells from the surface. Trophozoites and cysts were harvested at 500× *g* for 5 min and washed with PBS. For analysis of chitin production, cells were stained with 1 mL of PBS containing 10 μg/mL AlexaFluor WGA-488 for 15 min with constant rotation, rinsed with PBS, and fixed in 4% (*v:v*) paraformaldehyde in PBS for 15 min at ambient temperature. For analysis of nuclear content, sarkosyl-treated cysts or untreated trophozoites were ethanol-fixed and stained with propidium iodide as described above.

Fixed cells were washed twice in 1 mL PBS, resuspended in 1 mL PBS, and analyzed by flow cytometry using a CytoFLEX flow cytometer (Beckman Coulter Life Sciences, Indianapolis, Indiana). A 488 nm laser was used for excitation for both AlexaFluor WGA-488 and propidium iodide, and 100,000 events were collected for each sample. Flow cytometry data were analyzed using FCS Express 6 Flow. A uniform gating strategy was applied to all samples to determine trophozoite and cyst populations. Three biological replicates were used for each condition tested.

## 3. Results

### 3.1. Growth Conditions for Encystation

Encystation in *E. invadens* is routinely induced by growth at high cell density under nutrient stress in hypo-osmotic conditions. This is done by inoculating trophozoites at 500,000 cells per mL into diluted (47% concentration) TYI-S-33 medium lacking added glucose [26,27]. *E. invadens* cultures grown under these conditions are fully encysted within 3 days. We standardly culture *E. histolytica* HM1:IMSS trophozoites in TYI-S-33 medium containing 50 mM added glucose (TYI glucose medium) with 5000 cells per mL starting inoculum and passage cells into fresh medium every 3 days. In examining the effect of glucose deprivation on the growth of *E. histolytica*, daily observations of cultures inoculated at a higher starting cell density of 50,000 cells/mL in TYI-S-33 basal medium revealed the increased presence of cells with an unusual rounded morphology as growth progressed. These altered morphology cells were first observed at 2–3 days’ growth and became more prevalent over time, with cultures consisting predominantly of the altered cell form by 7 days. As the morphology and size of these cells resembled those of cysts, we set out to determine if these were indeed true cysts and whether it was possible to induce *E. histolytica* to encyst reproducibly in axenic culture by applying high starting cell density and nutrient stress.

For initial encystation trials with *E. histolytica*, trophozoites were inoculated at a starting cell density of 50,000 cells/mL into TYI basal medium, and cultures were examined microscopically every 24 h. Cell clumping, which is a characteristic of encystation in *E. invadens*, was visible as early as 48 h and was readily apparent by 72 h. Cells became rounder and decreased in size, and by 168 h, no live trophozoites were visible and only rounded cysts and dead cell debris remained. To ascertain that trophozoites had indeed undergone stage conversion to true cysts, we assessed the four hallmarks of encystation: presence of a chitin cell wall, tetranucleation, decreased cell size and roundedness, and detergent resistance [15,28,34,35].

### 3.2. Tetranucleation

*Entamoeba* trophozoites have a single nucleus, whereas mature cysts are tetranucleate [36,37]. As *E. histolytica* undergoes stage conversion within the colon, the nuclei begin dividing. Trophozoites that begin encysting during the G1/G0 phase of the cell cycle form cysts at a 1:1 ratio in which one trophozoite produces one cyst. Cells that begin encysting later in the progression of the cell cycle produce cysts at a 1:2 ratio [38]. Cells switch from the mitotic cycle into the developmental endopolyploid replication cycle in which DNA replication occurs without cell division, resulting in a tetraploid cyst [39]. Ganguly and Lohia [40] examined DNA content during growth and encystation in *E. invadens* in which they observed a decrease in 1n- and 2n-containing cells and a simultaneous increase in the number of 4n-containing cells as encystation progressed. They found that the DNA content of cysts varied from 4n to 8n or more. *E. histolytica* is frequently polyploid, with the DNA content of individual nuclei varying severalfold [38]. Although tetranucleation is considered the hallmark, mature *E. invadens* cysts have been observed with an even higher number of nuclei. Immature cysts or cyst-like structures have fewer than four nuclei [13,14,36,41].

**Figure 1:**
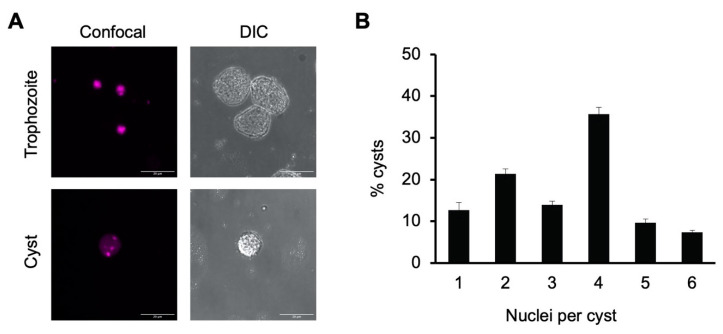
*E. histolytica* cysts are multinucleate. (**A**) Representative image of *E. histolytica* trophozoites (top) and sarkosyl-treated cysts (bottom) stained with propidium iodide for microscopic visualization of nuclei. Confocal images (left) and their corresponding DIC images (right) are shown with 20 μm scale bars. (**B**) Number of nuclei per cyst. Cysts from three biological replicates were stained with propidium iodide, and nuclei in 100 cysts from each culture were manually counted. Results shown are the mean ± standard deviation for three independent biological replicates.

Tetranucleation and increased DNA content in our in vitro cyst preparations were demonstrated using both confocal fluorescence microscopy and flow cytometry. Microscopic imaging of cells stained with propidium iodide was used to identify the number of nuclei in trophozoites grown in TYI glucose medium versus sarkosyl-treated cysts from week-old encystation cultures grown in TYI basal medium. Propidium iodide staining revealed the presence of a single nucleus in trophozoites but multiple distinct nuclei in cysts. Representative images of a stained cyst and trophozoite are shown in Figure 1A.

To assess the percentage of cysts with four or more nuclei, nuclei counts were performed on 100 cysts each from three biological replicates (Figure 1B). Slightly over half of all cysts had four or more nuclei present (52.3 ± 3.3% with 4–6 nuclei) and approximately two-thirds (66.3 ± 2.6%) had three or more nuclei. The average number of nuclei present was 3.3 ± 0.1. Although not all cysts were tetranucleate, this heterogeneity is consistent with previous reports that *E. invadens* cysts have an average of four nuclei and are a mixture of primarily tri- and tetranucleate cells [40,42].

### 3.3. Chitin Cell Wall

The presence of a chitin cell wall is a well-established hallmark of encystation in *Entamoeba* [43,44]. Chitin production has been one of the most extensively examined aspects of cyst formation and is the most readily measurable component of the encystation process. The chitin cell wall provides protection against environmental challenges faced by a cyst outside of the human host, including increased oxygen exposure, nutrient deprivation, and mechanical stressors. The major components of the *E. histolytica* cyst wall are chitin and the chitin-binding lectins Jacob, Jessie, and chitinase [45]. Each component is vital, and a “wattle and daub” model of the *Entamoeba* cyst wall was proposed wherein the chitin wall is crosslinked by the Jacob lectins and filled in with the Jessie lectins [44], creating an effectively impenetrable coat around the parasite. Without this protection, parasite transmission and survival would not be possible.

**Figure 2:**
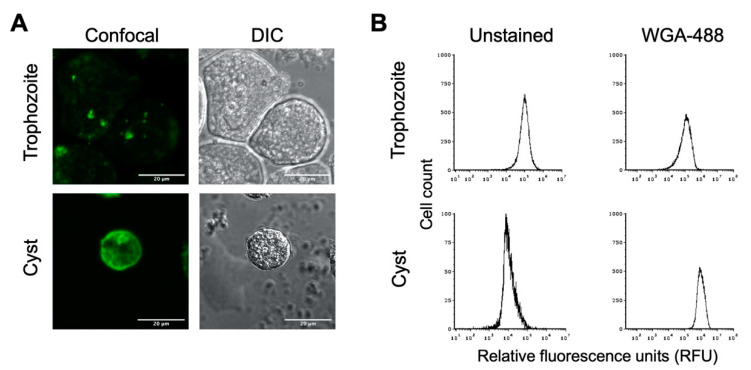
*E. histolytica* cysts have a chitin cell wall. *E. histolytica* trophozoites and sarkosyl-treated cysts were stained with AlexaFluor WGA-488 for microscopic visualization and flow cytometry analysis of the presence or absence of a chitin cell wall. (**A**) Representative images of *E. histolytica* trophozoites (top) and sarkosyl-treated cysts (bottom) stained with WGA-488. Confocal images (left) and their corresponding DIC images (right) are shown with 20 μm scale bars. (**B**) Flow cytometry analysis of WGA-488-stained trophozoites (top) and sarkosyl-treated cysts (bottom). Histograms on the left are from unstained cells, and those on the right are from cells stained with WGA-488. Histograms display cell count versus relative fluorescence units (RFUs) and are uniform for direct comparison of the position of the peaks. Three biological replicates were performed, and the results shown are representative.

To determine whether the in vitro cysts have a chitin cell wall, cyst preparations were treated with AlexaFluor WGA-488, a wheat germ agglutinin that binds to *N*-acetylglucosamine, the primary component of chitin. Confocal microscopic imaging revealed that *E. histolytica* cysts treated with WGA-488 had a fluorescent shell, indicating the presence of a chitin cell wall (Figure 2A). The uniformity of this fluorescent shell indicates a chitin cell wall fully encasing the cyst. As expected, no fluorescent shell was observed for *E. histolytica* trophozoites stained with WGA-488, indicating the absence of a chitin cell wall when *E. histolytica* is in its amoebic form. Similar results were observed when cysts were stained with Calcofluor White or Congo Red, both of which also bind to components of chitin.

Flow cytometry has previously been applied as a method for examining encystation in *Entamoeba* species [46,47], and we likewise used this to analyze *E*. *histolytica* trophozoite and cyst populations. Flow cytometry analysis of unstained trophozoite and cyst populations revealed distinct peaks of fluorescence, with trophozoites having higher autofluorescence than cysts (Figure 2B). Upon WGA-488 staining, the fluorescence intensity peak for the trophozoite population remained the same, indicating the absence of a chitin cell wall. The fluorescent peak for the cyst population was right-shifted to much higher fluorescence intensity than unstained cysts or WGA-488-treated trophozoites (Figure 2B). This is consistent with the presence of a chitin cell wall for cysts but not trophozoites and demonstrated that the flow cytometry of WGA-488-stained cells could be used to follow the progression into encystation on a population level. This peak of higher fluorescence intensity for WGA-488-stained cells is hereafter defined as the cyst peak (containing cells that are encysting as judged by the formation of a chitin cell wall), and the lower fluorescence intensity peak is defined as the trophozoite peak.

### 3.4. Small Size and Roundedness

As *E. histolytica* and *E. invadens* encyst, cell morphology is altered from an amoeboid form to a smaller, rounded morphology [6,15,28]. This notable change in shape and size coincides with increased expression of chitin synthase and is the result of the encasement in the chitinous cell wall [43]. We examined cyst size and morphology using both microscopy and flow cytometry. The representative DIC images of trophozoites and cysts shown in Figure 1A and Figure 2A clearly demonstrate the smaller size and rounded morphology of cysts in comparison with the larger, amorphously shaped trophozoites. The size of the cysts is consistent with the size range previously reported for *E. histolytica* and *E. invadens* cysts [6,15,28].

**Figure 3:**
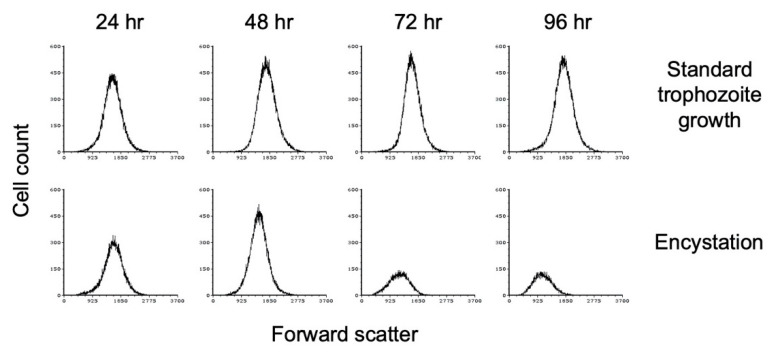
*E. histolytica* cysts are reduced in size versus trophozoites. Flow cytometry analysis was performed on WGA-488-stained *E. histolytica* cells during standard trophozoite growth (top) and encystation (bottom). Analysis was performed at 24 h intervals from 24 to 96 h and again at 168 h. Histograms display cell count versus forward scatter and are uniform for direct comparison of the position and size of the peaks. Three biological replicates were performed, and the results shown are representative.

Flow cytometry analysis has also been used to demonstrate that encystation is accompanied by a decrease in size as cells convert from trophozoite to cyst form [47]. We performed flow cytometry analysis of *E. histolytica* cultures grown at high cell density in TYI basal medium. Cultures from three biological replicates were harvested every 24 h for 96 h, stained with WGA-488, and subjected to flow cytometry analysis. As the cultures progressed through encystation, a decrease in forward scatter was observed (Figure 3), which is indicative of a decrease in size. Forward scatter decreased slightly by 48 h and continued to decrease over time. In comparison, low-density cultures grown in TYI glucose medium did not show any change in cell size (Figure 3), as would be expected for nonencysting trophozoites. The flow cytometry results also indicate that the cyst population has a more uniform size and shape. The total cell count in the later time points decreased, most likely indicating that a population of cells was unable to encyst and died.

### 3.5. Detergent Resistance

Like rounded shape, detergent resistance is also conferred by the chitin cell wall. The β-(1,4)-linked *N*-acetylglucosamine (GLnNAc) polymeric structure of the chitin cell wall provides resistance to harmful environmental stressors and resistance to disruption from strong detergents [48,49]. Cysts from *E. invadens* have been shown to be detergent resistant [48,49], which is considered a defining feature of true cysts. Treatment with 0.1% sarkosyl is the common method used to obtain uniform populations of *E. invadens* cysts [47].

**Figure 4:**
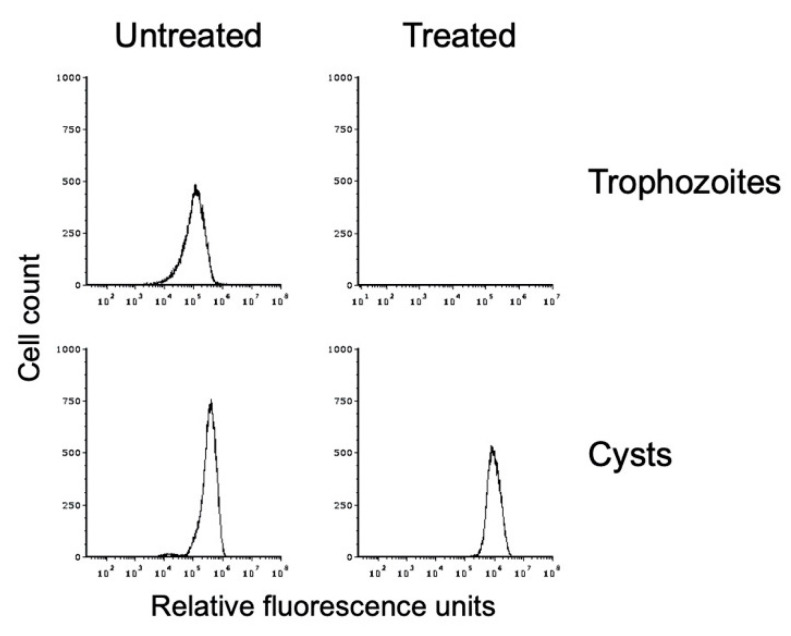
In vitro-formed *E. histolytica* cysts are detergent resistant. Flow cytometry analysis was performed on *E. histolytica* cells from standard trophozoite growth (top) and after encystation (bottom). Histograms from untreated cells are shown on the left and from sarkosyl-treated cells on the right. Histograms display cell count versus fluorescence intensity and are representative. Histograms are uniform for direct comparison of the position and size of the peaks. Three biological replicates were performed, and the results shown are representative.

We used sarkosyl treatment to demonstrate that our in vitro *E. histolytica* cysts were detergent resistant. Flow cytometry was performed on WGA-488-stained cyst preparations that had been split and either mock-treated or detergent-treated. This analysis demonstrated that the in vitro *E. histolytica* cysts were detergent resistant (Figure 4). A small reduction in the peak size and a shift to slightly higher fluorescence intensity were observed after detergent treatment, both of which may be due to lysis of immature cysts or cysts with a defective chitin cell wall. Overall, 82.3 ± 12.2% of cysts were detergent resistant over three biological replicates. Trophozoite samples that were likewise split and mock-treated or sarkosyl-treated demonstrated that detergent treatment resulted in complete lysis of the population (Figure 4), with <0.1% of the population remaining after detergent treatment in all three biological replicates. DIC imaging of sarkosyl-treated cysts revealed no signs of damage, and the chitin cell wall remained intact. These results are consistent with the formation of an intact chitin cell wall that provides protection to the cyst.

### 3.6. Viability and Excystation

We used an esterase viability assay to demonstrate that the in vitro-formed *E. histolytica* cysts are fully viable. Cyst viability was examined using the fluorescein diacetate (FDA) hydrolysis assay, in which FDA is hydrolyzed to fluorescent fluorescein by live cells but not dead cells [13,33]. The viability was shown to be 96.3 ± 1.2% for cysts produced in TYI basal medium using this assay.

Cyst viability was also demonstrated by their ability to excyst to the growing trophozoite form. Cysts from 1-week encystation cultures were detergent-treated and inoculated into standard TYI glucose medium in 12-well culture plates, in triplicate, and the plates were sealed and incubated at 37 °C for 5 days. Daily microscopic observations were performed, and trophozoites were harvested at 5 days post-inoculation and counted. Microscopic observation revealed the presence of the first few trophozoites by 24 h post-inoculation, and trophozoites became prevalent by 48 h. By day 5, the cultures were nearly confluent, with 8.9 ± 1.7 × 10^5^ trophozoites per mL, representing a minimum of nine doublings for each cyst that excysted to trophozoite form. These results demonstrate that our method for inducing encystation of *E. histolytica* in laboratory culture produced viable cysts that are capable of stage conversion back to growing, dividing trophozoite form.

Cultures of trophozoites produced by excystation of in vitro cysts were able to encyst once again (I. Bastuzel, personal communication), demonstrating that completion of the developmental cycle between trophozoite and cyst and back is sustainable in cysts produced by the methods described here. Optimization of excystation conditions and determination of environmental signals for excystation will be investigated separately and are beyond the scope of this study.

### 3.7. Role of Cell Density in Encystation

Having established that viable cysts that exhibited all four of the established hallmarks of true cysts were formed, we evaluated the effect of different environmental signals on encystation. Signals that induce differentiation into either stage are necessary to maintain infection, through both maintenance of parasite numbers within the host and propagation of infection to other hosts [15]. Stress conditions, including glucose deprivation, high cell density, and osmotic stress, have been shown to be important signals for encystation in *E. invadens* in vitro [15,26,27,28,50].

We first observed *E. histolytica* cells with cyst morphology after 7–10 days in cultures inoculated at a low cell density of ~5000 cells/mL in TYI basal medium but not in cultures grown in TYI glucose medium. In cultures inoculated at a higher cell density of 50,000 trophozoites/mL in TYI basal medium, cells exhibiting cyst morphology were apparent by ~48 h and became prevalent by 72 h. These observations and the fact that encystation of *E. invadens* is routinely elicited by culturing trophozoites at a very high starting cell density of 500,000 trophozoites/mL in diluted TYI basal medium suggest that cell density and nutrient deprivation play a role in eliciting encystation [26,27]. Cell aggregation, mediated by gal-terminated ligands, is reported to be a prerequisite for encystation in *Entamoeba* parasites [51], so it is perhaps not surprising that high cell density plays a role.

To investigate the impact of cell density on encystation, we inoculated TYI basal medium at different cell densities and followed entry into encystation by monitoring chitin cell wall formation by flow cytometry. Replicate cultures for three biological replicates were inoculated at starting cell densities of 10,000, 25,000, 50,000, and 100,000 trophozoites/mL into TYI basal medium (designated hereafter as 10K basal, 25K basal, 50K basal, and 100K basal cultures). One culture for each biological replicate of each starting cell density was harvested every 24 h up to 96 h and then at 168 h when cultures were expected to be fully encysted. Harvested cells were stained with WGA-488 and analyzed by flow cytometry. A uniform gating strategy was applied to all flow cytometry analyses.

**Figure 5:**
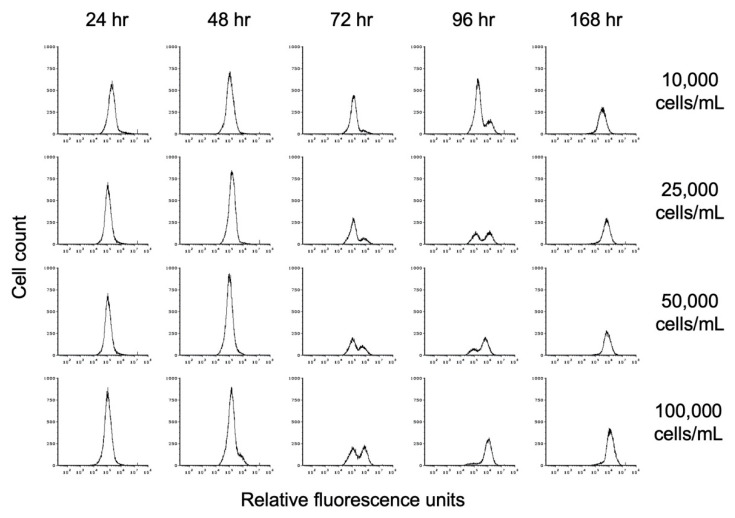
Flow cytometry analysis of the effect of starting cell density on encystation. Replicate *E. histolytica* cultures inoculated at 10,000, 25,000, 50,000, or 100,000 trophozoites/mL into TYI basal medium were harvested at 24, 48, 72, 96, and 168 h, stained with WGA-488, and analyzed by flow cytometry. Histograms display cell count versus relative fluorescence units and are uniform for direct comparison of the position and size of the peaks. Three biological replicates were performed, and the results shown are representative.

The progression toward encystation was monitored by the disappearance of the lower fluorescence intensity (left) trophozoite peak and the appearance of the higher fluorescence intensity (right) cyst peak, indicating the deposition of the chitin cell wall during encystation. The histograms shown in Figure 5 demonstrate the progression of encystation over time at different starting cell densities. The results from one of the three biological replicates are shown. The rate of appearance and increase in size of the cyst peak corresponds to starting cell density, with the cyst peak appearing earlier in cultures with higher starting cell density than in cultures with lower starting cell density. As the size of the cyst peak increased, that of the trophozoite peak decreased. By 168 h, only the cyst peak was present regardless of starting cell density.

**Figure 6:**
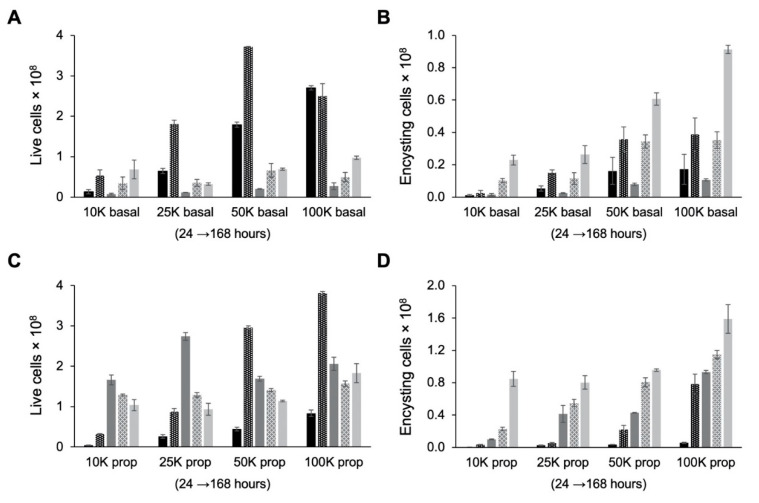
Effect of cell density on growth and encystation in TYI basal medium. Replicate *E. histolytica* cultures inoculated at 10,000 (10K), 25,000 (25K), 50,000 (50K), or 100,000 (100K) trophozoites/mL into TYI basal (**A** and **B**) or TYI propionate medium (**C** and **D**) were harvested at 24, 48, 72, 96, and 168 h (left to right for each set of cultures), stained with WGA-488, and analyzed by flow cytometry. (**A**) Live cell count in TYI basal cultures. (**B**) Percentage of live encysting cells in TYI basal cultures. (**C**) Live cell count in TYI propionate cultures. (**D**) Percentage of live encysting cells in TYI propionate cultures. Values are the mean ± standard deviation for three biological replicates.

Examination of live cell counts after cells were transferred from TYI glucose medium to TYI basal medium for encystation revealed that cultures experienced severe (85–95%) die-off between the 48 and 72 h time points (Figure 6A). This was presumably due to nutrient deprivation as fresh medium is not supplied as during culture maintenance. Examination of encysting cells, defined as live cells in the high fluorescence intensity peak, revealed a similar reduction in cell count between 48 and 72 h post-inoculation (Figure 6B). The number of encysting cells began to increase again after 72 h. The final encystation efficiency, defined as the percentage of live cells in the high fluorescence intensity peak at 168 h post-inoculation, ranged from ~60% for the 10K basal cultures to ~90% for the 100K basal cultures (Table 1). The lower final encystation efficiency observed for the 10K basal cultures may be because these cultures had not yet completed growth and encystation and with additional time might reach a similarly high encystation efficiency as observed with the other starting cell densities.

The final number of cysts produced by 168 h (Table 1) was two- to threefold higher than the number of live cells remaining after this die-off, indicating that subpopulation that lived continued to proliferate slowly and progress toward encystation. Presumably, these cells either had adapted their metabolism to better use other nutrients in the medium or were able to scavenge nutrients from the dead cells. Surprisingly, although cultures inoculated at a higher starting cell density produced the most cysts, the rate of encystation, defined as the final number of cysts divided by the starting number of trophozoites, was highest for cultures with the lowest starting cell density (Table 1).

Although encystation of *E. invadens* in culture was reported as early as 1962 [52], the effect of cell density on encystation was not investigated until 1984 when Vazquezdelara-Cisneros and Arroyo-Begovich found that starting cell density affected both the time at which initial signs of encystation were observed and final cyst yield [27]. Increased starting cell density lowered the time of appearance of encysting cells to as early as 7 h post-inoculation at the highest starting cell densities of 5 × 10^5^ to 1 × 10^6^ cells/mL. The highest cyst yield was observed with 5 × 10^5^ cells/mL but decreased at 1 × 10^6^ cells/mL starting inoculum.

Our results indicate that encystation is asynchronous, as the increase in the higher fluorescence intensity cyst peak was accompanied by a decrease in the lower fluorescence intensity trophozoite peak, and this shift was gradual over the 1-week time period. This is not surprising, as encystation in the human colon must also be asynchronous, with only a subpopulation of trophozoites entering encystation at any given time. This allows colonization and infection to be maintained even as infected persons shed millions of cysts daily [9,15,28]. This can occur if only a subpopulation of trophozoites colonizing the colon progresses to encystation at any given time. Cell density may thus play a key role in regulating encystation in vivo by allowing encystation to occur only when a certain threshold density of cells has been achieved and by maintaining that threshold level to ensure that colonization continues.

### 3.8. Effect of Propionate on Encystation

As short-chain fatty acids (SCFAs) are prevalent in the large intestine due to the metabolic activity of the gut microbiome [53,54], we examined their effect on encystation in *E. histolytica*. The total SCFA concentration is ~100–120 mM [54] with a relative ratio of 57:22:21 for acetate, propionate, and butyrate, respectively, for all regions of the large intestine [53]. Acetate has been proposed to be absorbed through the intestinal wall to the portal vein, and the colon epithelium metabolizes butyrate [53,55], making propionate perhaps the most readily available SCFA for utilization by *E. histolytica*. Therefore, in our initial studies we focused on the effect of propionate on encystation because we found in trial experiments that the addition of propionate to TYI basal medium increased *E. histolytica* growth unlike acetate or butyrate (T. Dang and C. Ingram-Smith, personal communication).

We produced cysts in TYI basal medium containing 24 mM sodium propionate final concentration (hereafter designated as TYI propionate medium) using a standard starting cell density of 50,000 cells/mL and 168 h incubation period. This concentration of propionate was chosen based on the total SCFA concentration and the relative ratio given above. We measured viability using the esterase assay and found that 97.3 ± 0.9% of cysts were viable. These cysts were also able to excyst back to growing trophozoites when placed in TYI glucose medium, as for cysts produced in TYI basal medium. In addition, we performed nuclei counts and found that the cysts produced in TYI propionate medium had a similar distribution to that observed for cysts produced in TYI basal medium, with 58.9 ± 4.5% cysts having 4–6 nuclei (36.6 ± 1.5% with exactly 4 nuclei) and an average of 3.6 ± 0.2 nuclei per cyst across the entire population for the three biological replicates. Thus, the cysts produced in the presence of propionate appear to indeed be true cysts.

**Figure 7:**
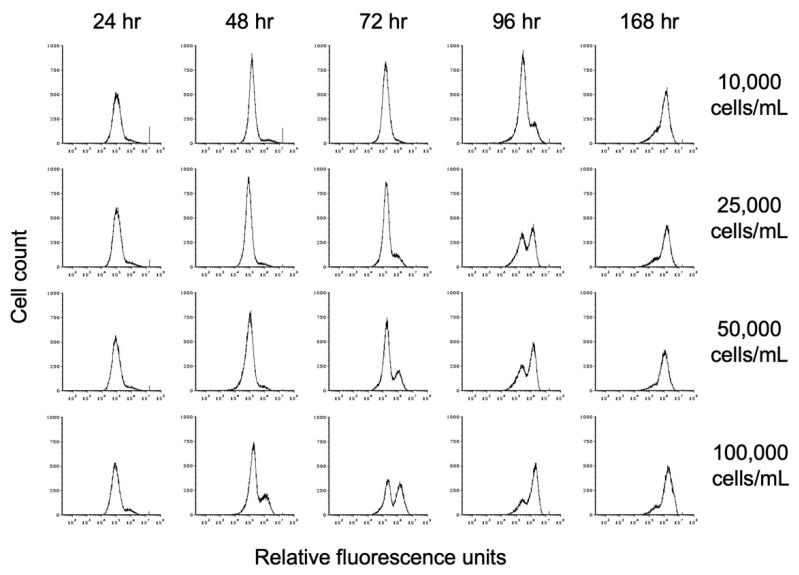
Effect of propionate on encystation. Replicate *E. histolytica* cultures inoculated at different cell densities into TYI propionate medium were harvested at 24 h intervals, stained with WGA-488, and analyzed by flow cytometry. Histograms display cell count versus relative fluorescence units and are uniform for comparison of the position and size of the peaks. Three biological replicates were performed, and the results shown are representative.

To investigate the impact of propionate on encystation, we inoculated TYI propionate medium at starting cell concentrations of 10,000, 25,000, 50,000, and 100,000 trophozoites/mL (hereafter designated as 10K prop, 25K prop, 50K prop, and 100K prop). Cell count and entry into encystation were monitored over time by flow cytometry as described above. Representative flow cytometry histograms showing the time course of encystation in TYI propionate at different cell densities are shown in Figure 7.

Examination of live cell count revealed that TYI propionate cultures did not experience the same die-off as observed for the TYI basal cultures (Figure 6C). TYI propionate cultures experienced a lesser die-off starting between the 72 and 96 h time points and continuing through the 168 h time point. Additionally, unlike the case for encystation in TYI basal medium, the number of encysting cells increased steadily throughout the entire 168 h period (Figure 6D). This resulted in a higher number of cysts produced in TYI propionate medium versus TYI basal medium at all starting cell densities (Table 1). When cyst production was normalized versus starting cell number, the encystation rate for the 10K basal and 10K prop cultures was significantly higher than that for the respective 25K, 50K, and 100K cultures. The results also showed that the encystation rate was significantly higher in the presence versus absence of propionate across all starting cell densities (Table 1). Overall, comparison of the results observed in the absence versus presence of propionate indicates that propionate has a pronounced effect on encystation and greatly increases the number of cysts produced and the overall rate of encystation.

### 3.9. Effect of Other SCFAs on Encystation

**Figure 8:**
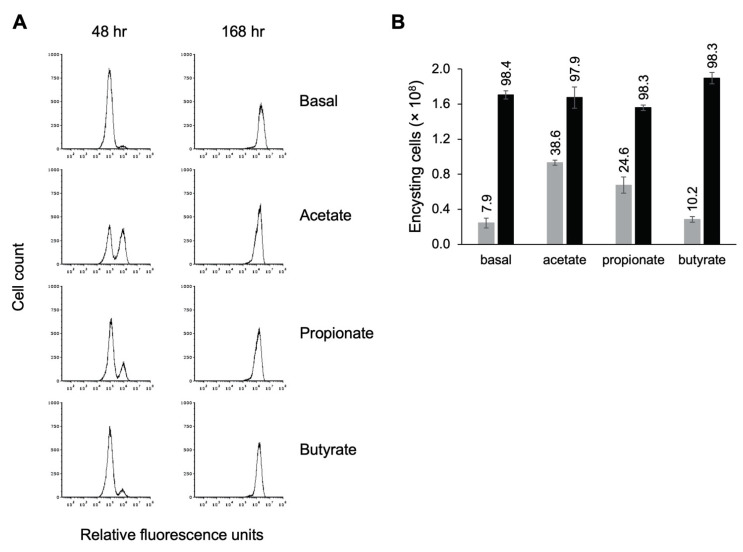
Short-chain fatty acids speed entry into encystation. Replicate *E. histolytica* encystation cultures grown in TYI basal medium or TYI basal medium supplemented with 62 mM acetate, 24 mM propionate, or 23 mM butyrate were harvested at 48 and 168 h post-inoculation, stained with WGA-488, and analyzed by flow cytometry. (**A**) Representative histograms display cell count versus relative fluorescence units and are uniform for comparison of the position and size of the peaks. Three biological replicates were performed, and the results shown are representative. (**B**) Total number of encysting cells and cysts at 48 h (gray bars) and 168 h (black bars) post-inoculation. Values are the mean ± standard deviation for three biological replicates. The percentage of live encysting cells is indicated above each bar.

Having demonstrated that the presence of propionate resulted in earlier entry into encystation, we were interested in whether other SCFAs influenced encystation as well. Visual inspection of TYI basal cultures supplemented with acetate indicated that cells entered encystation earlier, as judged by the appearance of cells with the small, rounded morphology of cysts. To examine this more closely, cultures were inoculated at 50,000 trophozoites/mL in TYI basal medium or TYI basal medium containing 62 mM acetate, 24 mM propionate, or 23 mM butyrate, representing concentrations similar to those found in the colon [53]. Cells were harvested, stained with WGA-488, and analyzed by flow cytometry to determine how many cells had begun encysting by 48 h and the overall final number of cysts produced by 168 h. Representative histograms are shown in Figure 8A, and quantitative results from three biological replicates are shown in Figure 8B.

We found that addition of acetate had the strongest effect on entry into encystation, followed by propionate (Figure 8A). In TYI acetate cultures, 38.6% of live cells had begun forming a chitin cell wall by 48 h versus 24.6% for TYI propionate cultures (Figure 8B). These values were statistically different from that observed for TYI basal cultures (7.9%), with *p*-values of 0.0002 and 0.0015 for TYI acetate and TYI propionate, respectively. There was no statistical difference between TYI basal and TYI butyrate cultures. By 168 h, all of the cultures had fully encysted. Although the addition of acetate and propionate appeared to induce earlier entry into encystation, the overall final level of encystation was generally unaffected by the addition of SCFAs as similar numbers of cysts were formed by 168 h under all four conditions (Figure 8B). Whether this earlier entry into encystation was acetate specific or due to the higher concentration of acetate versus propionate was not investigated. The observation that propionate enhanced early entry into encystation but butyrate did not even though both were added at similar concentrations suggests that it is the SCFA used rather than the concentration that is important.

Our results showing that acetate and propionate influence the initiation of encystation in *E. histolytica* contrast with those for *E. invadens*. Byers et al. [56] found that the addition of butyrate to 10 mM final concentration in TYI basal medium had no effect on the growth of *E. invadens*, and the addition of acetate, propionate, and butyrate at a 70:20:10 mM ratio slightly inhibited growth. The presence of SCFAs inhibited *E. invadens* encystation in encystation medium (TYI basal medium diluted to 47% concentration) in a dose-dependent manner, with propionate and butyrate having a more potent effect than acetate. The presence of all three SCFAs at a final concentration of ~6 mM total SCFA at a 70:20:10 ratio (acetate/propionate/butyrate) inhibited encystation by 50%, and full inhibition was observed at normal physiological concentrations (~100 mM total final concentration). SCFA concentrations in herbivorous reptiles have been shown to be 71–77 mM acetate, 12–14 mM propionate, and 8–12 mM butyrate [57,58,59], which are a similar range to those observed in humans. This suggests that the signals for encystation only partially overlap for *E. histolytica* and *E. invadens*.

## 4. Conclusions

We have developed the first reproducible method for encystation of *E. histolytica* in laboratory culture that produces viable cysts exhibiting all four hallmarks of true cysts. Although the reptile pathogen *E. invadens* has been used as a model for encystation, the genetic and environmental differences between *E. histolytica* and *E. invadens* are a major limitation. This was demonstrated directly by our finding that short-chain fatty acids enhanced entry into encystation for *E. histolytica* but were previously shown to inhibit encystation of *E. invadens*. The ability to produce *E. histolytica* cysts in an in vitro system is a critical research advancement that now allows us to investigate stage conversion, which plays a critical role in disease transmission, directly in the human pathogen.

## Figures and Tables

**Table 1:** Cyst production, encystation efficiency, and encystation rate.

Starting Cell Density (cells/mL)	TYI Basal	TYI Propionate	*p*-Value: Basal vs. Propionate
	Total cysts produced (× 10^7^)		
10K	2.29 ± 0.30	8.48 ± 0.93	*p* = 0.0004
25K	2.63 ± 0.55	8.04 ± 0.84	*p* = 0.0007
50K	6.07 ± 0.39	9.57 ± 0.16	*p* = 0.0001
100K	9.13 ± 0.25	15.90 ± 1.77	*p* = 0.0028
	Encystation efficiency (% cysts/live cells)		
10K	33.5 ± 4.3	81.5 ± 8.9	*p* = 0.0011
25K	81.7 ± 17.0	86.1 ± 9.0	NS
50K	87.6 ± 5.6	83.9 ± 1.4	NS
100K	93.8 ± 2.6	86.9 ± 9.7	NS
	Encystation rate (total cysts/total starting cell population)		
10K	44.0 ± 5.7	163.0 ± 17.8	*p* = 0.0004
25K	20.2 ± 4.2	61.8 ± 6.5	*p* = 0.0007
50K	23.3 ± 1.5	36.8 ± 0.6	*p* = 0.0001
100K	17.6 ± 0.5	30.6 ± 3.4	*p* = 0.0028

## Data Availability

Data presented in this study are all available within the article.
